# Lettuce romaine calm and manage our glycemia: adding leafy greens to a meal may improve postprandial metabolism

**DOI:** 10.1186/s12944-021-01495-9

**Published:** 2021-07-15

**Authors:** Gregory C. Henderson

**Affiliations:** Department of Nutrition Science, Purdue University, 700 West State Street, West Lafayette, IN 47907 USA

When considering lifestyle factors such as consumption of fruits and vegetables, and leading a physically active lifestyle, the benefits are often ascribed to the long-term, chronic effects of each lifestyle factor. An example of this long-term mindset would be views of antioxidant biology; long-term intakes of anti-oxidants could potentially prevent the accumulation of oxidative damage to DNA and other cellular constituents that accumulate over months and years. However, it is important to note that lifestyle factors (e.g., nutrition) typically have both acute and chronic effects. The acute effects of nutrient intake can be manifested as a modification of postprandial metabolism following the individual meal in which that specific food was consumed. With a substantial portion of each day spent in the postprandial state, any effects of diet selection on postprandial metabolism could exert major health impacts. It is a well-accepted goal to maintain blood glucose and blood lipids within desirable ranges over the course of each day, and these factors respond to the components of each individual meal. Following each mixed meal, circulating levels of glucose and triacylglycerol (TAG) rise and then fall. Each rise contributes to the subject’s lifetime total exposure of blood vessels and tissues to these metabolites. The acute effects of each meal accumulate over the years. While the majority of nutrition research in the past has been focused on chronic intake of foods, the acute effects of consuming specific foods within a meal are also important for understanding health properties of nutrition.

Including specific foods into a meal may modify the stresses imposed upon metabolism following that meal. For example, as discussed above, glucose and lipoprotein responses to a meal could be modified by specific foods or food groups. General effects of dietary fiber and macronutrient composition in test meals upon blood glucose and TAG have been elucidated previously [4, 10]; however, significant gaps in knowledge remain. To translate our understanding to real-world application, a greater level of information is needed in relation to the intake of complex dietary components that simultaneously deliver digestible macronutrients, fiber, micronutrients, and trace levels of other important biologically active compounds. Specific foods, such as leafy green vegetables, might have impacts that are beyond those which we would predict based upon our current understanding. To develop knowledge about the benefits of specific foods that are likely to be healthful, acute studies of specific foods are needed, such as specific leafy greens and other vegetables.

It is of public health interest that blood glucose and TAG rise in individuals following consumption of each mixed meal, because postprandial responses of blood glucose [5] and TAG [1] are risk factors for developing metabolic and cardiovascular diseases. Thus, developing new approaches to blunt the responses is desirable. In the current issue of *Lipids in Health and Disease*, Shokraei and colleagues [12] performed a randomized, controlled, cross-over trial to investigate acute responses to three different meals. In this study of healthy young men, the authors administered test meals to study the acute responses of blood glucose and lipoproteins. The work of Shokraei et al. is important for advancing the field forward, and it will be advantageous to the scientific community if a greater number of such translational studies of acute nutrition are conducted in the future by investigators around the world. In the study by Shokraei et al. [12], each meal contained approximately 800 kcal of energy with a macronutrient composition that somewhat reasonably matches that of a typical daily intake, with the majority of energy from carbohydrate (CHO), a substantial proportion of energy from fat, and the remaining balance from protein (~ 50% CHO, ~ 40% fat, ~ 10% protein). On each of 3 occasions, participants consumed the meal with addition of romaine lettuce, watercress, or inclusion of cellulose (fiber) to the control meal in place of the leafy green vegetables. Blood was drawn periodically over 4 h following each meal for tracking of blood glucose and lipoproteins. As compared with the fiber-containing control meal, addition of these leafy greens did not significantly alter the responses of circulating TAG or other components of the lipoprotein profile. However, the postprandial blood glucose response was blunted when lettuce was consumed with the meal, in comparison to meals containing watercress or fiber supplement. While blood glucose rose following the meals containing cellulose or watercress, when the meal contained lettuce blood glucose actually trended downward in the postprandial period. This divergent response in the lettuce trial led to a significant main effect of the meal in the statistical analysis, indicating that the post-meal blood glucose profile was lower in the lettuce-containing meal. It is noteworthy that the response to romaine lettuce could not be recapitulated by addition of a different leafy green (watercress) or by fiber intake alone. Thus, the benefits of romaine lettuce intake upon glycemic control may be unique to this specific food, and it is unknown how generalizable this discovery is to other types of vegetables and other types of lettuce. The findings are encouraging and point to a need for additional research.

The work by Shokraei and colleagues is an excellent example of translational science. In translational research, it is ideal to develop a study design that incorporates aspects of the real-world experience of humans, in order to maximize clinical relevance. The authors have achieved this goal by testing specific leafy vegetables within a well-controlled test meal. They have tested real foods that one may select for inclusion in meals in daily life, yet they conducted the research in a laboratory setting with application of useful biochemical measurements. Thus, the work was translational as it was within the spectrum from basic science to clinical research (or from “bench to bedside”), incorporating aspects of basic science laboratory control as well as aspects of real-world meal consumption. Often in translational studies of postprandial metabolism, physical activity on the day of experimentation is rigidly controlled. For example, to exclude potential impacts of physical activity when studying nutrition, often subjects would be asked to remain nearly motionless for the entire day. When physical activity is limited in this situation, the trials would be scheduled on days when students need not attend class or when employed individuals need not report to their jobs. However, Shokraei and colleagues took a different approach and chose to allow greater levels of physical activity in the participants. The research subject population included students, and subjects were permitted to leave the laboratory between blood draws to attend classes [12]. The authors report that approximately 40% of the subjects did walk to classes between blood draws. Allowing participants to carry out these activities reflects the translational nature of the study, as it makes the study design similar to a normal day in the life of the participants. However, while this lack of physical activity control perhaps makes the results applicable to the real world, it is also a potential weakness of the study. It is known from previous publications that a recent bout of exercise can reduce blood glucose and plasma TAG for hours [3, 7, 8]. While the subjects did not exercise at any intense level, one could postulate that bouts of walking might have some similar effect, yet to a lesser extent. Indeed, it has been reported that even brief bouts of walking spaced throughout the day, to break up sedentary sitting behavior, can reduce blood glucose concentrations [2, 6, 9, 11]. Thus, permitting subjects to leave the laboratory on foot between blood draws in the study by Shokraei et al. could have led to reduced blood glucose levels. Nonetheless, the authors report that physical activity patterns were similar between the lettuce, watercress, and fiber control trials; therefore, it is expected that the findings for effects of lettuce upon blood glucose are valid, yet it should be noted that the benefits of lettuce were observed on the background of the potential benefits of physical activity in nearly half of the participants. This mixture of possible physical activity effects and food effects upon metabolism is of course what occurs in real daily life, and it might be of interest in the future to further explore any interactions between physical activity and vegetable intake upon postprandial blood glucose levels. Clearly, the study by Shokraei and colleagues paves the way for future work on other dietary components, clinical populations, and potential interactions between diet and physical activity. Their study represents a valuable step forward in our understanding of dietary patterns and health. Additionally, as is often the case with impactful research, the study leads to many questions that will require future studies to fill the remaining knowledge gaps.

The study by Shokraei and colleagues exemplifies the notion that healthy foods need not be consumed regularly, for many months, in order to achieve initial health benefits. While long-term intake of leafy greens is certainly advisable, inclusion of certain leafy vegetables (such as romaine lettuce) in even a single meal can improve the postprandial metabolic milieu on that occasion through acute effects upon metabolism. The cumulative effect of the 24-h integrated blood glucose and lipid levels, over many months and years, ultimately exerts a meaningful impact on one’s disease risk; a promising approach for reducing this risk is to focus upon improving the postprandial response to each individual meal by including foods which exert acute effects upon postprandial metabolism. Discovering ways to alter the response of glycemia to each meal would be of high clinical value, such that over the years an individual experiences a lower total exposure of blood vessels to glucose. Thus, the authors are commended for performing a valuable study of the acute responses to nutrition. This study [12] is very encouraging and is supportive of the concept that foods can exert their benefits through acute effects upon metabolism immediately following consumption. Studying romaine lettuce has led to important new knowledge, and the findings may inspire future work on other potentially healthful components of the diet. Considering the vast number of leafy greens and other vegetables available to consumers, it is clear that the present results about lettuce may only be the tip of the iceberg.

## Data Availability

Not applicable.

## References

[1] Bansal S, Buring JE, Rifai N, Mora S, Sacks FM, Ridker PM (2007). Fasting compared with nonfasting triglycerides and risk of cardiovascular events in women. Jama.

[2] Brocklebank LA, Andrews RC, Page A, Falconer CL, Leary S, Cooper A (2017). The acute effects of breaking up seated office work with standing or light-intensity walking on interstitial glucose concentration: a randomized crossover trial. J Phys Act Health.

[3] Davitt PM, Arent SM, Tuazon MA, Golem DL, Henderson GC (2013). Postprandial triglyceride and free fatty acid metabolism in obese women after either endurance or resistance exercise. J Appl Physiol (1985).

[4] Desmarchelier C, Borel P, Lairon D, Maraninchi M, Valero R. Effect of nutrient and micronutrient intake on chylomicron production and postprandial lipemia. Nutrients. 2019;11(6):1299. 10.3390/nu11061299. 10.3390/nu11061299 PMC6627366 31181761

[5] Expert Panel on Detection E and Treatment of High Blood Cholesterol in A (2001). Executive summary of the third report of the National Cholesterol Education Program (NCEP) expert panel on detection, evaluation, and treatment of high blood cholesterol in adults (Adult Treatment Panel III). JAMA.

[6] Freire YA, Macedo GAD, Browne RAV, Farias-Junior LF, Bezerra ADL, Fayh APT, Farias Junior JC, Boreskie KF, Duhamel TA, Costa EC (2019). Effect of breaks in prolonged sitting or low-volume high-intensity interval exercise on markers of metabolic syndrome in adults with excess body fat: a crossover trial. J Phys Act Health.

[7] Henderson GC, Fattor JA, Horning MA, Faghihnia N, Johnson ML, Luke-Zeitoun M, Brooks GA (2008). Glucoregulation is more precise in women than in men during postexercise recovery. Am J Clin Nutr.

[8] Henderson GC, Krauss RM, Fattor JA, Faghihnia N, Luke-Zeitoun M, Brooks GA (2010). Plasma triglyceride concentrations are rapidly reduced following individual bouts of endurance exercise in women. Eur J Appl Physiol.

[9] Ma SX, Zhu Z, Zhang L, Liu XM, Lin YY, Cao ZB (2020). Metabolic effects of three different activity bouts during sitting in inactive adults. Med Sci Sports Exerc.

[10] O'Keefe JH, Gheewala NM, O'Keefe JO (2008). Dietary strategies for improving post-prandial glucose, lipids, inflammation, and cardiovascular health. J Am Coll Cardiol.

[11] Rodriguez-Hernandez M, Martin JS, Pascoe DD, Roberts MD, Wadsworth DW (2018). Multiple short bouts of walking activity attenuate glucose response in obese women. J Phys Act Health.

[12] Shokraei S, Khandouzi N, Sina Z, et al. The acute effect of incorporating lettuce or watercress into a moderately high-fat meal on postprandial lipid, glycemic response, and plasma inflammatory cytokines in healthy young men: a randomized crossover trial. Lipids Health Dis. 2021;20:66. 10.1186/s12944-021-01487-9. 10.1186/s12944-021-01487-9 PMC8281573 34261489

